# Therapeutic effect of SP‐8356 on pulmonary embolism‐associated cardiac injury is mediated by its ability to suppress apoptosis and inflammation

**DOI:** 10.1111/jcmm.16535

**Published:** 2021-05-04

**Authors:** Jia Zhou, Kai Liu, Cheng Feng, Zhengliang Peng, Wei Luo

**Affiliations:** ^1^ Department of Emergency The First Affiliated Hospital of South China University Hengyang China; ^2^ Department of Pharmacology The Central Hospital of Hengyang Hengyang China; ^3^ Department of Cardiovascular Medicine The First Affiliated Hospital of South China University Hengyang China

**Keywords:** apoptosis, Cyclophilin A‐CD147, ERK1/2, inflammation, MMP‐9, P65, pulmonary embolism‐associated cardiac injury, SP‐8356

## Abstract

The cyclophilin A–CD147 interaction has been reported to be one of the most potential therapeutic targets for the treatment of acute pulmonary embolism. The signalling of extracellular signal‐regulated kinase 1/2 (ERK1/2) was also reported in the pathogenesis of cardiac injury. Since SP‐8356 is regarded as a novel Inhibitor of CD147‐Cyclophilin, the study aimed to evaluate potential therapeutic effects of SP‐8356 for pulmonary embolism‐associated cardiac injury. Western blot and immunohistochemistry were carried out to analyse the expression of MMP‐9, ERK1/2, phosphorylated ERK1/2 (p‐ERK1/2), P65, p‐P65, and CyA protein in PE cell and rat models under distinct conditions. Flow cytometry and TUNEL were carried out to examine the apoptosis of primary rat myocardiocytes and PE rat models under distinct conditions. CyA treatment on primary rat myocardiocytes remarkably raised the expression of MMP‐9, p‐ERK1/2 and p‐P65 protein expression; SP8536 treatment effectively restored the CyA‐induced up‐regulation of MMP‐9, p‐ERK1/2 and p‐P65 protein expression in primary rat myocardiocytes. Besides, flow cytometry analysis showed that SP8536 remarkably suppressed the CyA‐induced elevation of cell apoptosis rate of primary rat myocardiocytes. Moreover, SP8536 notably diminished the abnormal elevation of right ventricular systolic pressure (RVSP), Troponin I and Myeloperoxidase activity in PE rat models. Furthermore, SP‐8536 significantly restored the up‐regulation of MMP‐9, p‐ERK1/2, p‐P65, CyA protein and the cellular apoptosis in the PE rat model. Our study validated that SP‐8356 could suppress cell apoptosis and inflammatory response via down‐regulating the highly expressed MMP‐9, p‐ERK1/2, and p‐P65 and MMP‐9 in PE‐associated cardiac injury in a dose‐dependent manner.

**Abbreviations**
PE: pulmonary embolism
RV: right ventricular
RVSP: right ventricular systolic pressure

## 1 INTRODUCTION

Acute pulmonary embolism (APE) 2 is one of the typical and most dangerous illnesses with a 30‐day average death rate of ~10%.^1^ The existence of right ventricular (RV) disorder is correlated to a considerable rise in hospitalization and mortality due to pulmonary embolism (PE).^1^, ^2^ Prior research shows that an inflammatory response is a crucial factor in the dysfunction of the right ventricle after APE. Large amounts of matrix metalloproteinases (MMPs) are released and activated after APE related inflammatory cells flow into to right ventricular cells.^1^, ^3^, ^4^ Activated matrix metalloproteinases (particularly MMP‐2 and MMP‐9) might deteriorate myosin light chain‐1, cardiac troponin‐I (cTnI) and several other sarcomeric cytoskeletal proteins, thus causing myocardial contractile disorder related to APE.^5^, ^6^ Several scientific studies have revealed that anti‐inflammatory substances or the inhibition of matrix metalloproteinases attenuate heart muscle injury and RV dysfunction in models of APE.^7^, ^8^

Recently discovered small molecule, SP‐8356(( 1S,5R) −4‐( 3,4‐dihydroxy‐5‐methoxystyryl) −6,6‐ dimethylbicyclo [3.1.1] hept‐3‐en‐2‐one), is a verbenone by‐product reducing inflammation and oxidation.^9^ Additionally, it has been recently discovered that mechanism of SP‐8356 involves binding to CD147 and reduction in neointimal hyperplasia via MMP‐9 inhibition.^10^ This activity also contributes to anti‐tumour activity of SP‐8356.^11^

Cyclophilin A (CyPA) comprises 0.1%–0.6% of all the cytosolic healthy proteins and play multiple cellular functions like immunomodulation, cell signalling, transcription regulation, protein folding and trafficking.^12^, ^13^ Over the last decade, researchers have illustrated that in response to inflammatory stimulations such as hypoxia, infection and oxidative stress CyPA might be released from the cells.^12^, ^14^ CyA interacts with its cell‐surface signalling receptor cluster of differentiation 147 (CD147), additionally called extracellular matrix metalloproteinase inducer (EMMPRIN), extracellular CyPA can trigger ERK/nuclear factor (NF)‐ B paths(pathways), stimulate cytokine release, speed up leukocyte recruitment and also improve MMP activation at the site of injury.^12^, ^13^, ^15^ CD147 is a crucial factor in the process of inflammation.^12^, ^16^, ^17^ Targeted inhibition of CD147 by either small interfering siRNA or monoclonal antibody (mAb) induces MMP‐9 thus, exert profound anti‐atherosclerotic effects both in vitro and in vivo.^16^, ^18^ Communication between CyPA and CD147 causes acute or chronic inflammation in several diseases.^12^, ^13^, ^19^, ^20^ Inhibition of CypA‐CD147 interaction relieves myocardial inflammation, remodelling in troponin I‐induced myocarditis, and decreases infarct size after myocardial reperfusion and ischaemia.^21^ The result of the study clearly showed that SP‐8356 indirectly suppresses the MMP pathway via inhibition of CD147 and exhibiting an anti‐atherosclerotic effect. The apoptosis rate in heart cells in the icariin and an icariin � CD147 groups was considerably low as compared to in that model group.

The interaction between cyclophilin A and CD147 has been reported to present a potential therapeutic target for the treatment of APE,^22^ while the signalling of ERK1/2 was also reported by previous studies to be associated with the pathogenesis of cardiac injury.^11^, ^23^ Since SP‐8356 is regarded as a novel Inhibitor of CD147‐Cyclophilin, our study therefore aims to investigate the potential therapeutic application of SP‐8356 for PE‐associated cardiac injury.^11^, ^24^

## 2 MATERIALS AND METHODS

### 2.1 Animal models and treatment

A total of 36 adult male Sprague‐Dawley rats (weighing approximately 300‐320 g, purchased from Taconic Biosciences, New York) were randomly divided into four groups as follows: 1. a sham group, 2. an APE control group, 3. an APE plus SP‐8356 (low‐dose) group and 4. an APE plus SP‐8356 (high‐dose) group. For the low‐dose SP‐8356 group, SP‐8356 was injected by a daily i.p. administration at the dosage of 10 mg/kg. And for the high‐dose SP‐8356 group, SP‐8356 was injected by a daily i.p. administration at the dosage of 50 mg/kg. And for the APE procedure, all rats were tethered to a workbench following anaesthetization by 80 mg/kg sodium pentobarbital via i.p. injection. The right femoral vein was cannulated under a stereomicroscope, and the experimental model of APE was created by injecting a suspension of microspheres (Sephadex G‐50, dia. 300 µm, Thermo Fisher Scientific) into the inferior vena cava at the dosage of 12 mg/kg. Similarly, to create a sham group, an equivalent amount of normal saline instead of microspheres was injected into the rats. The dose of microsphere was established based on preliminary experiments and previous publications.^22^ Plasma samples were collected from all animal groups. The right ventricle tissue samples were excised and processed, respectively, with 10% formalin (for immunofluorescence assay) or stored at 70°C (for Western blot assay). All experiments were carried out under NIH’s Guide for the Care and Use of Laboratory Animals and were approved by the institutional ethics committee.

### 2.2 Cell culture and transfection

The samples of cardiomyocyte tissue were excised from the right ventricles of the SD rats and treated with trypsin (0.03%), collagenase (0.03%) and DNase I (20 µg/mL). The cells were cultured in a Dulbecco's Modified Eagle Medium: Nutrient Mixture F‐12 medium supplemented with 10% FBS and penicillin‐streptomycin maintained at 37°C, saturated humidity as well as 5% carbon dioxide. In this study, primary rat myocardiocytes were randomly divided into four groups: 1. Negative Control group, 2. CypA (200 ng/mL) group (primary rat myocardiocytes were treated with 200 ng/mL CypA, which was obtained from Abcam), 3. CyA (200 ng/mL)+SP‐8356 (1 μM) group (primary rat myocardiocytes were first treated with 1 µM SP‐8356 and later treated with 200 ng/mL of CypA), and 4. CyA (200 ng/mL)+SP‐8356 (5 μmol/L) group (primary rat myocardiocytes were treated with 5 uM SP‐8356 followed by 200 ng/mL of CypA). The dosage of CyA and SP‐8356 was confirmed according to previous published studies.^25^ Cells were rinsed with PBS (pH = 7.4) and blocked with a normal goat serum (Thermo Fisher Scientific, MA), followed by incubation with an anti‐Cyclophilin A antibody (Thermo Fisher Scientific, MA) for 30 minutes at 4°C and goat anti‐Rabbit IgG (H + L) Cross‐Adsorbed Secondary Antibody, Alexa Fluor 488 (Thermo Fisher Scientific, MA) as the secondary antibody.

### 2.3 Western blot analysis

The ability of SP‐8356 to inhibit CyPA‐induced expression and corresponding protein levels of MMP‐9, ERK1/2, p‐ERK1/2, P65 and p‐P65 in primary rat myocardiocytes was assessed using Western blotting. The samples were added to the RIPA buffer, homogenized and centrifuged. The supernatant layer was collected. Protein levels were measured by the bicinchoninic acid kit (Thermo Fisher Scientific). The proteins extracted from the primary rat myocardiocytes were electrophoresed in SDS‐PAGE using the Invitrogen apparatus (Thermo Fisher Scientific) at a constant voltage. In the next step, the separated proteins were blotted to a PVDF membrane (EMD Millipore) and blocked using 5% skim milk, and the blot was incubated with specific primary antibodies against MMP‐9, ERK1/2, p‐ERK1/2, P65 and p‐P65. As a secondary antibody, a Horseradish peroxidase goat anti‐mouse immunoglobulin (BD Bioscience) was used. The protein bands were developed using the enhanced chemiluminescence (ECL) reagent (Invitrogen).

### 2.4 Apoptosis analysis

The primary rat myocardiocytes were divided into four groups as above: 1. Negative Control group, 2. CypA (200 ng/mL) group, 3. CyA (200 ng/mL)+SP‐8356 (1 μmol/L) group and 4. CyA (200 ng/mL)+SP‐8356 (5 μM) group. The primary rat myocardiocytes were carefully treated with CyA with or without the pre‐treatment of SP‐8356 (1 or 5 μmol/L) for 24 h, and then incubated for 30 minutes with a combination of Annexin V and propidium iodide solution (Thermo Fisher Scientific) at 37°C, which helps to differentiate between the necrotic and apoptosis cells. Using Flowing Software (Turku Bioscience), the apoptotic fractions of the cells were analysed.

### 2.5 Immunohistochemistry

The tissue microarray blocks were used for immunohistochemistry staining. Briefly, the TMA blocks were immersed in 10 mmol/L of the EDTA buffer and reacted at 125°C for 10 minutes to retrieve the MMP‐9, p‐ERK1/2 and p‐P65 antigen. After 3% H_2_O_2_ in MeOH treatment and 3% BSA treatment, the slides were then incubated with antibodies against MMP‐9, p‐ERK1/2 and p‐P65 (Abcam) in cold temperature for a day. The IHC stains for the MMP‐9, p‐ERK1/2 and p‐P65 proteins were produced and a counterstain of haematoxylin was applied.

### 2.6 ELISA

Myeloperoxidase activity was quantified in plasma samples with an ELISA kit (Abcam, Cambridge, UK). All samples were run in triplicate, and the sample mean value is expressed as pmol plasma.

### 2.7 TUNEL assay

TUNEL assay was carried out according to the manufacturer's instructions (TUNEL kit, Beyotime, Shanghai, China). Briefly, the cells, divided into four groups: 1. a sham group, 2. a PE group, 3. PE plus SP‐8356 (1 µmol/L) and 4. PE plus SP‐8356 (5 µmol/L), were washed with PBS, and the antigen retrieval was performed using a citrate buffer. Later, the slices were washed, 50 µL of peroxidase‐labelled anti‐digoxigenin was added to the slices. Finally, DAPI solution was added and incubated for 10 minutes. After that, the fluorescence microscope was observed.

### 2.8 Statistical analysis

All experiments were carried out ≥three times. Data were shown as Mean ± SD. Comparisons among multiple groups were done using one‐way ANOVA in SPSS 19.0 (IBM) software. Tukey's test was used as the post hoc test of one‐way ANOVA. *P* values of ≤0.05 were taken into consideration as statistically significant.

## 3 RESULTS

### 3.1 SP‐8536 significantly restored the CyA‐induced up‐regulation of MMP‐9, p‐ERK1/2, p‐P65 and elevation of cell apoptosis rate of primary rat myocardiocytes

**FIGURE 1:**
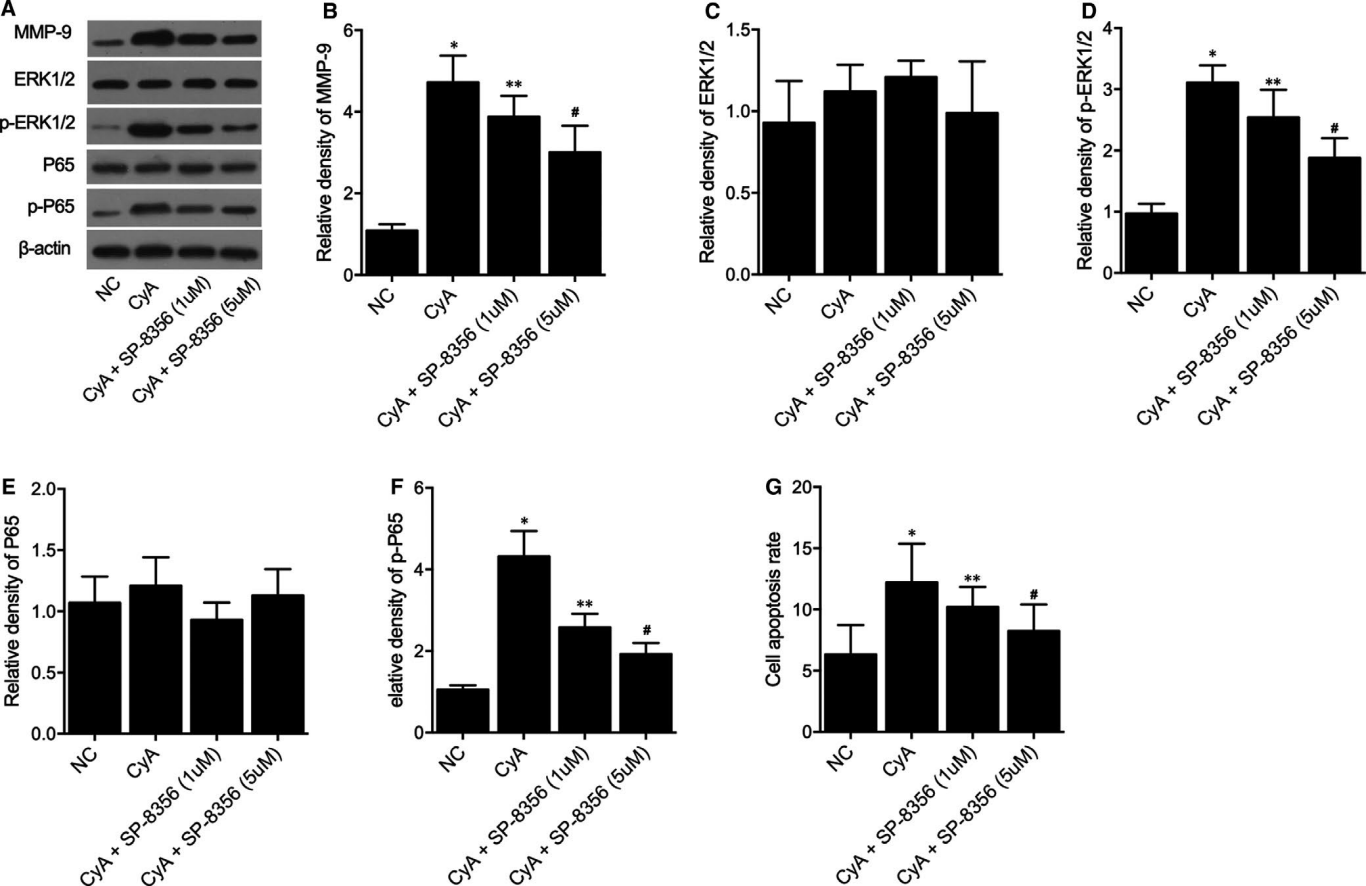
SP‐8536 significantly restored the CyA‐induced up‐regulation of MMP‐9, p‐ERK1/2, p‐P65 and elevation of cell apoptosis rate of primary rat myocardiocytes (*P value < 0.05 vs. NC group; **P value < 0.05 vs. CyA group; and # *P* value < 0.05 vs. CyA+SP‐8356 (1 μmol/L) group). A, Western blot analysis showed differential expression of MMP‐9, ERK1/2, p‐ERK1/2, P65 and p‐P65 protein in primary rat myocardiocytes under CyA and SP‐8536 treatment. B, The CyA‐induced up‐regulation of MMP‐9 was decreased by SP‐8536 in a dose‐dependent manner. C, No obvious difference was observed for the expression of ERK1/2 in primary rat myocardiocytes under distinct conditions. D, The CyA‐induced up‐regulation of p‐ERK1/2 was decreased by SP‐8536 in a dose‐dependent manner. E, No obvious difference was observed for the expression of P65 in primary rat myocardiocytes under distinct conditions. F, The CyA‐induced up‐regulation of p‐P65 was decreased by SP‐8536 in a dose‐dependent manner. G, The CyA‐induced elevation of cell apoptosis was decreased by SP‐8536 in a dose‐dependent manner

Primary rat myocardiocytes were treated with CyA followed by SP‐8356 treatment; Western blot was performed on primary rat myocardiocytes under distinct conditions to evaluate the expression of MMP‐9, ERK1/2, p‐ERK1/2, P65 and p‐P65 protein (Figure 1A). The expression of MMP‐9 in primary rat myocardiocytes was increased by CyA treatment; SP‐8536 reversed the CyA effect MMP‐9 up‐regulation in a dose‐dependent manner (Figure 1B). No obvious difference was found for the expression of ERK1/2 under distinct conditions (Figure 1C). However, the expression of p‐ERK1/2 was increased by CyA treatment and SP‐8536 reversed the CyA‐induced p‐ERK1/2 up‐regulation in a dose‐dependent manner (Figure 1D). Similarly, no obvious difference was found for the expression of P65 under distinct conditions (Figure 1E). But SP‐8536 effectively reversed in a dose‐dependent manner the CyA‐induced p‐P65 up‐regulation (Figure 1F). Moreover, flow cytometry was carried out to observe the cell apoptosis rate of primary rat myocardiocytes under distinct conditions, the cell apoptosis rate was significantly elevated by CyA, SP‐8536 restored the CyA‐induced elevation of primary rat myocardiocytes apoptosis in a dose‐dependent manner (Figure 1G).

### 3.2 SP‐8536 maintained the RVSP, Troponin I and myeloperoxidase activity in PE rat models

**FIGURE 2:**
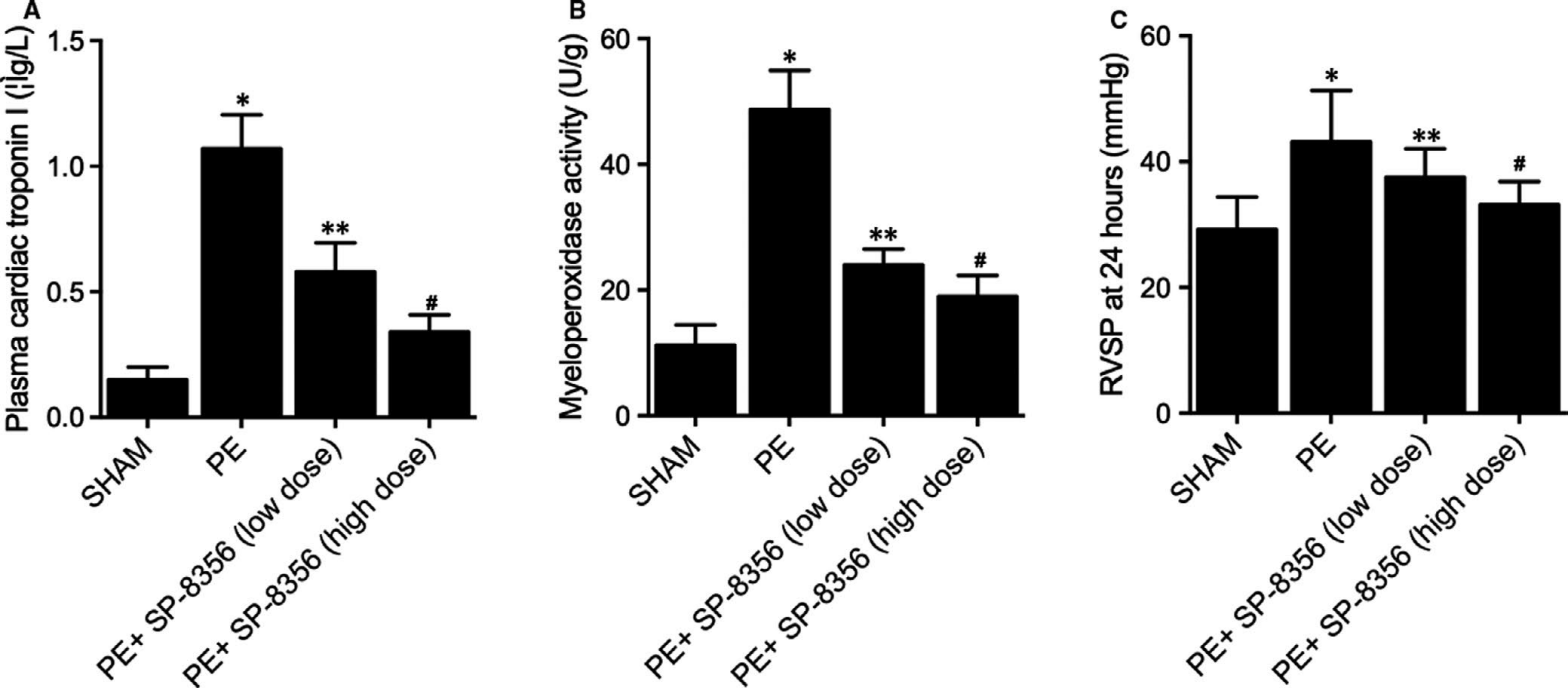
SP‐8536 maintained the RVSP, Troponin I and myeloperoxidase activity in PE rat models (*P value < 0.05 vs. SHAM group; **P value < 0.05 vs. PE group; and # *P* value < 0.05 vs. PE+SP‐8356 (low‐dose) group). A, The increased RVSP in PE rat model was restored by SP‐8536 in a dose‐dependent manner. B, The increased Troponin I in PE rat model was restored by SP‐8536 in a dose‐dependent manner. C, The increased Myeloperoxidase in PE rat model was restored by SP‐8536 in a dose‐dependent manner

To gain a deep insight into the mechanism of the therapeutic efficiency of SP‐8536 on PE, a PE rat model was established and subjected to SP‐8536 treatment at a low dose and high dose. The RVSP of PE rat was remarkably elevated when compared with the control, SP‐8536 treatment notably decreased the RVSP in the PE rat model in a dose‐dependent manner (Figure 2A). Besides, the Troponin I and Myeloperoxidase activity in plasma was further examined in PE rat models under distinct conditions. The elevated cardiac troponin I (Figure 2B) and myeloperoxidase activity (Figure 2C) in the plasma of the PE rat model were effectively restored by SP‐8536 treatment in a dose‐dependent manner.

### 3.3 SP‐8536 significantly restored the up‐regulation of MMP‐9, p‐ERK1/2, p‐P65 and CyA protein in the PE rat model

**FIGURE 3:**
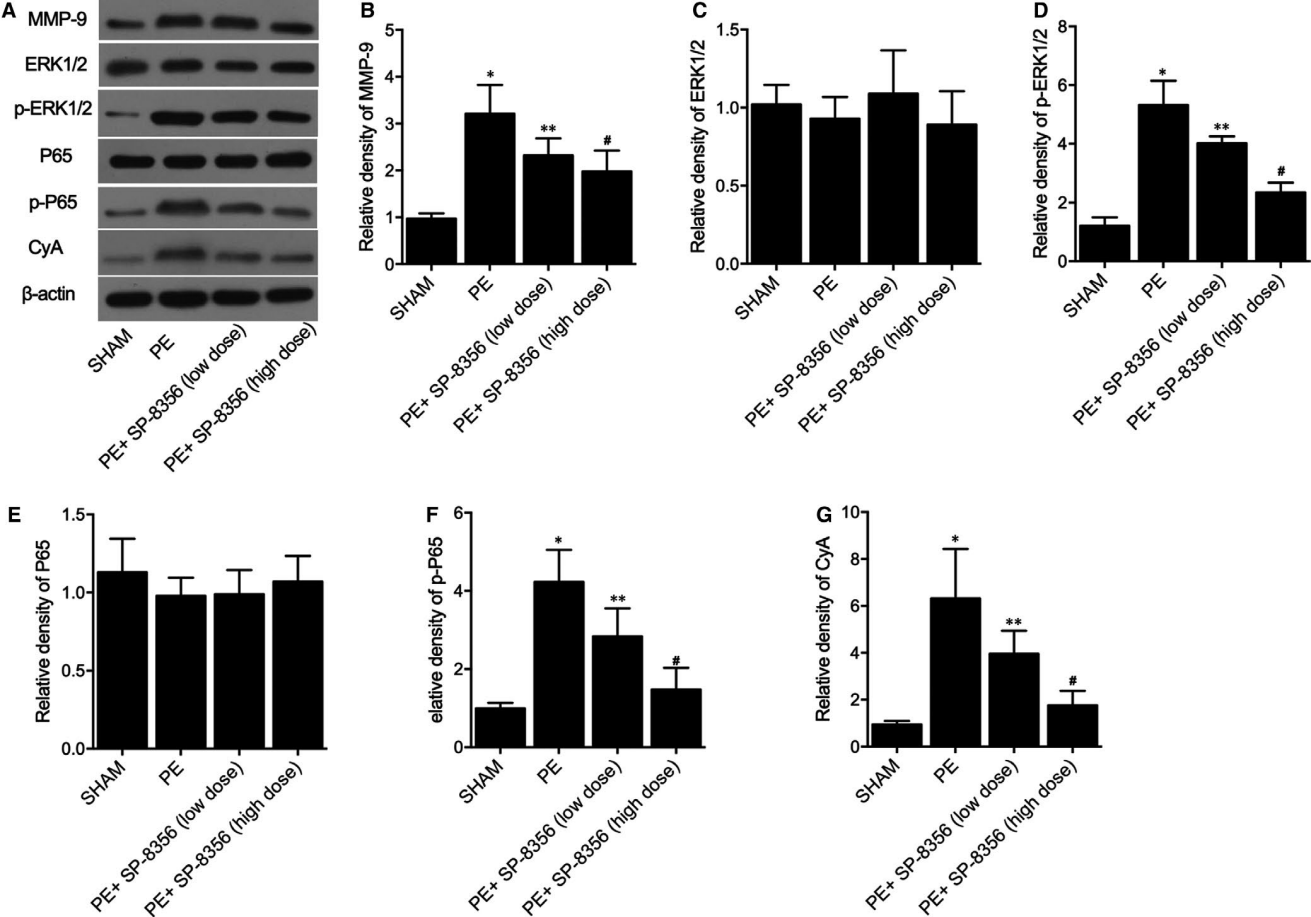
SP‐8536 significantly restored the up‐regulation of MMP‐9, p‐ERK1/2, p‐P65 and CyA protein in PE rat model (*P value < 0.05 vs. SHAM group; **P value < 0.05 vs. PE group; and #*P* value < 0.05 vs. PE+SP‐8356 (low‐dose) group). A, Western blot analysis showed differential expression of MMP‐9, ERK1/2, p‐ERK1/2, P65, p‐P65 and CyA protein in PE rat models under distinct conditions. B, SP‐8536 decreased the elevated expression of MMP‐9 in PE rat models in a dose‐dependent manner. C, No obvious difference was observed for the expression of ERK1/2 in PE rat models under distinct conditions. D, SP‐8536 decreased the elevated expression of p‐ERK1/2 in PE rat models in a dose‐dependent manner. E, No obvious difference was observed for the expression of P65 in PE rat models under distinct conditions. F, SP‐8536 decreased the elevated expression of p‐P65 in PE rat models in a dose‐dependent manner. G, SP‐8536 decreased the elevated expression of CyA in PE rat models in a dose‐dependent manner

Furthermore, Western blot was carried out to analyse the distinct expression of MMP‐9, ERK1/2, p‐ERK1/2, P65, p‐P65 and CyA protein in PE rat models under differential conditions (Figure 3A). The expression of MMP‐9, p‐ERK1/2, p‐P65 and CyA was significantly increased in the PE rat model; SP‐8536 treatment effectively restored the elevation of MMP‐9 (Figure 3B), p‐ERK1/2 (Figure 3D), p‐P65 (Figure 3F) and CyA (Figure 3G) protein expression in PE rat models. However, the expression of ERK1/2 (Figure 3C) and P65 (Figure 3E) showed no obvious changes under differential conditions.

### 3.4 SP‐8536 significantly restored the elevation of apoptosis of PE rat models

**FIGURE 4:**
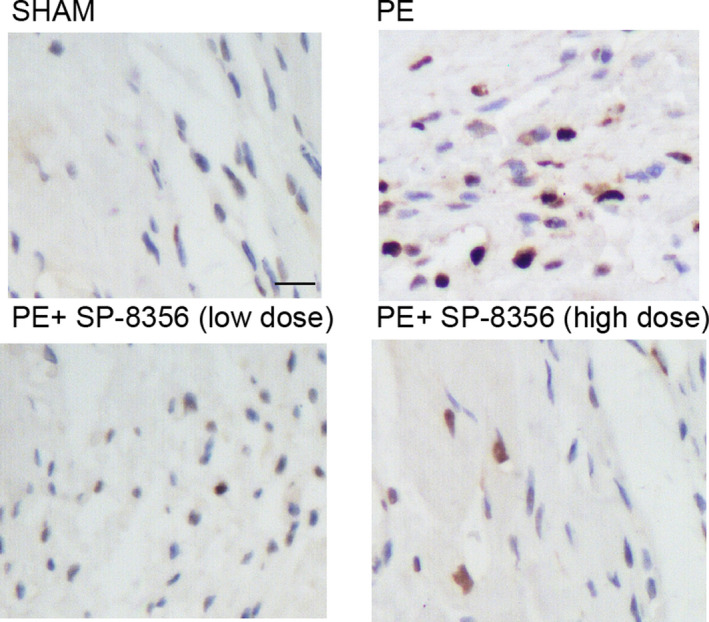
TUNEL analysis showed that SP‐8536 significantly restored the elevation of apoptosis of PE rat models (scale bar: 50 μm)

TUNEL was performed to analyse the apoptosis of the PE rat model under low‐dose and high‐dose treatment of SP‐8536. The apoptosis was remarkably elevated in PE rat models when compared with the control, SP‐8536 showed evident efficiency in decreasing the elevated apoptosis of PE rat models (Figure 4).

### 3.5 IHC analysis indicated SP‐8536 significantly restored the up‐regulation of MMP‐9, p‐ERK1/2, p‐P65 and CyA protein in the PE rat model

**FIGURE 5:**
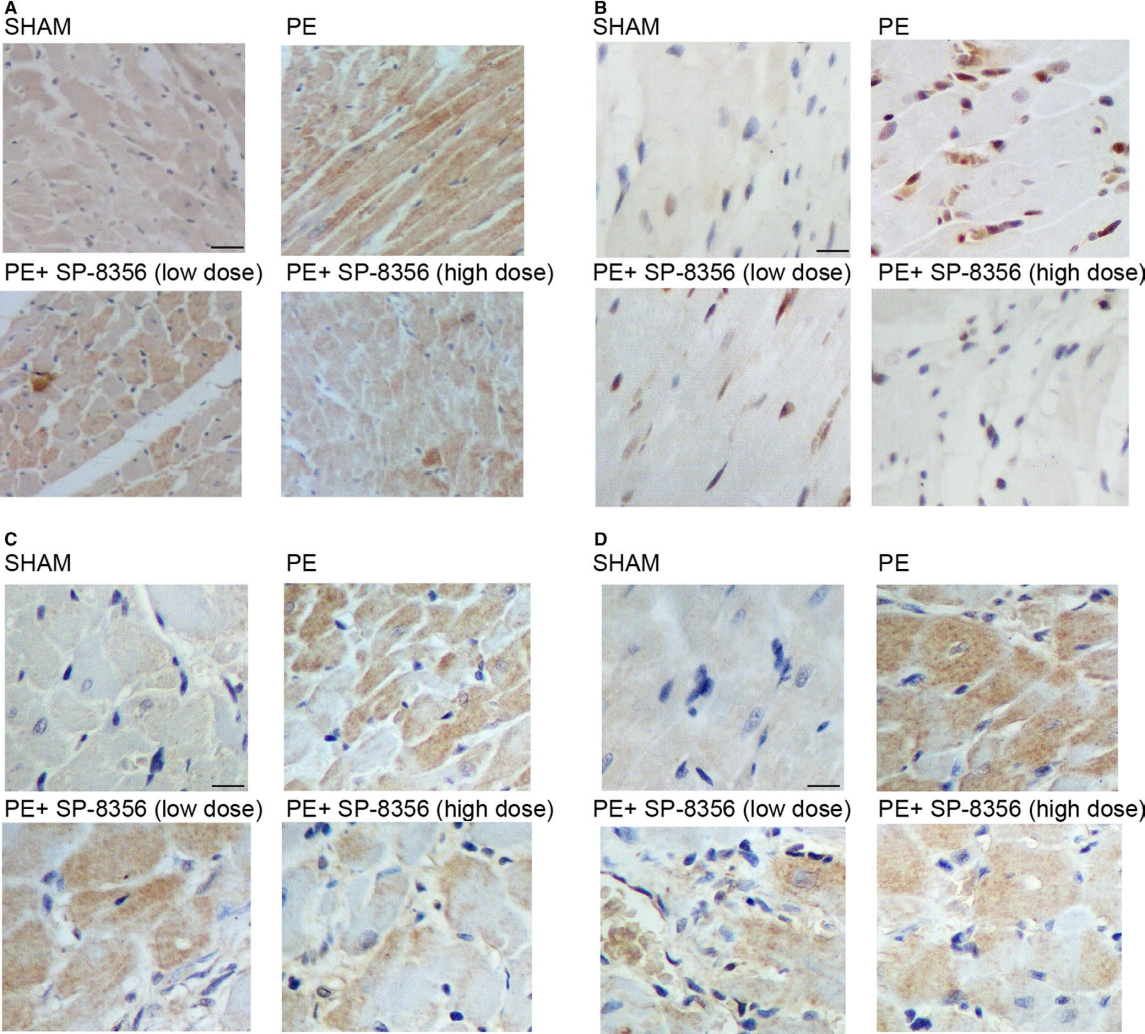
IHC analysis indicated SP‐8536 significantly restored the up‐regulation of MMP‐9, p‐ERK1/2, p‐P65 and CyA protein in PE rat model (scale bar: 50 μm). A, SP‐8536 decreased the elevated expression of MMP‐9 in PE rat models in a dose‐dependent manner. B, SP‐8536 decreased the elevated expression of p‐ERK1/2 in PE rat models in a dose‐dependent manner. C, SP‐8536 decreased the elevated expression of p‐P65 in PE rat models in a dose‐dependent manner. D, SP‐8536 decreased the elevated expression of CyA in PE rat models in a dose‐dependent manner

Immunohistochemistry was used to further analyse the distinct expression of MMP‐9, p‐ERK1/2, p‐P65 and CyA protein in PE rat models under differential conditions. Based on the observation in Western blot assay and IHC analysis, the expressions of MMP‐9, p‐ERK1/2, p‐P65 and CyA were significantly increased in the PE rat model, SP‐8536 treatment effectively restored the elevation of MMP‐9 (Figure 5A), p‐ERK1/2 (Figure 5B), p‐P65 (Figure 5C) and CyA (Figure 5D) protein expression in PE rat models.

## 4 DISCUSSION

A novel small molecule therapy, SP‐8356, has potential activity against inflammation and oxidative stress.^9^ It blocks enlargement of neointimal tissues and also maintaining plaque susceptibility in animal models of PE via the inhibition of MMP‐9 function. Anti‐tumour activity of SP‐9356 is well proven by the previous studies.^10^, ^11^ SP‐8356 likewise inhibits transcriptional activity of NF‐κB, which generates the expression of genes in charge of cell proliferation, survival, as well as mobility. The growth inhibitory effect of SP‐8356 was slightly more than that of JSH‐23 and another inhibitor of NF‐κB transcriptional activity (information is not shown). In this study, we performed Western blot and IHC to analyse the expression of MMP‐9, ERK1/2, p‐ERK1/2, P65, p‐P65, and CyA protein in PE cell and rat models. SP‐8536 significantly restored the up‐regulation of MMP‐9, p‐ERK1/2, p‐P65, and CyA protein in PE cell and rat models.

Inflammatory response provoked by APE produces heart muscle injury and arrhythmia in the RV of the heart. The study also suggests that treatment of CypA partially activated ERK1/2 kinase NF‐κB pathway. This leads to the reduction in the accumulation of neutrophil cells, and subdued activation of MMP‐9 and MMP‐2 in RV cardiomyocytes, subsequently exacerbating RV injury and disorder after APE. Combination therapy of CypA and inhibition of its cellular receptor in animal studies attenuated both heart muscle cell injury and dysfunction of right ventricle compared to stand‐alone therapy by either CypA or CD147.

In mouse models, the pathophysiology of CVB3‐activated myocarditis involves several Inflammatory processes (a) up‐regulation of CyPA and its extracellular receptor CD147, (b) CyPA is crucial for the recruitment of macrophages and T cells along with the defence against the virus during the early stages of CVB3 infection, and (c) ability of CsA analog, NIM811, to inhibit cyclophilin and provides protection against myocardial fibrosis, can be measured based on the collagen tissues in the chronic myocarditis during CVB3 infection. The level of CyPA is elevated in RV cells of patients suffering from APE; therefore, blocking the interaction between CyPA and its extracellular receptor can protect against myocardial injury, and RV dilation and dysfunction. Along with the above‐mentioned beneficial effects of modulation of the CyPA and its extracellular receptor interactions following APE, these were also related to a lower level of myeloperoxidase activity and lowered MMP‐9 and MMP‐2 levels. Activation of the ERK1/2 and p38 MAPK signalling pathways following binding of the secreted CyPA increases cell proliferation via both autocrine and paracrine mechanisms. In this study, we carried out flow cytometry and TUNEL to evaluate the apoptosis of PE cell and rat models under distinct conditions, respectively. SP‐8536 showed the strong capability to suppress the elevation of apoptosis rate in PE cell and rat models in a dose‐dependent manner.

The results of this study clearly show that following SP‐8536 therapy, various phosphorylated proteins were elevated including p‐NF‐κB, P65 and p‐P65 in IECs. Even though the levels of p‐NF‐κB and p‐P65 were elevated, the overall expression of NF‐κB and P65 remained unaffected. These results suggested a deeper insight into the mechanism of therapeutic effect of SP‐8536. Previous study showed that dual therapy of infliximab and methotrexate was effective in reducing CD147 expression on the CD14� monocytes. The combination therapy also reduced levels of MMP‐9 mRNA in peripheral blood mononuclear cells and the serum levels of both MMP‐3 and MMP‐9 compared to the serum levels of patients treated with methotrexate monotherapy.^12^

Matrix metalloproteinases (MMPs) are a class of enzymes responsible for the deterioration of components of the EM. The primary findings of this research study were as follows: (a) the levels of serum cardiac troponin I am positively correlated to the progress of APT; (b) the concentrations of plasma pro‐MMP‐9 raise dramatically in late‐stage APT; and (c) the prognosis of APT is positively correlated to the above‐mentioned biomarkers. In this study, we used ELISA to check the Myeloperoxidase activity in PE rat models under distinct conditions, the elevation of Myeloperoxidase activity was effectively suppressed by SP‐8536 in a dose‐dependent manner.

## 5 CONCLUSION

As a result, our study validated that SP‐8356 could suppress cell apoptosis and inflammatory response via down‐regulating the highly expressed MMP‐9, p‐ERK1/2, and p‐P65 and MMP‐9 in PE‐associated cardiac injury in a dose‐dependent manner.

## CONFLICT OF INTEREST

None.

## AUTHOR CONTRIBUTIONS

**Jia Zhou:** Conceptualization (equal); Data curation (equal); Investigation (equal); Methodology (equal); Resources (equal). **Kai Liu:** Data curation (equal); Investigation (equal); Software (equal); Visualization (equal). **Cheng Feng:** Investigation (equal); Methodology (equal); Visualization (equal). **Zhengliang Peng:** Formal analysis (equal); Investigation (equal); Software (equal); Validation (equal); Writing‐original draft (equal). **Wei Luo:** Funding acquisition (equal); Project administration (equal); Resources (equal); Supervision (equal); Writing‐original draft (equal).

## Data Availability

The data that support the findings of this study are available from the corresponding author upon reasonable request.
